# Gene duplication and fragmentation in the zebra finch major histocompatibility complex

**DOI:** 10.1186/1741-7007-8-29

**Published:** 2010-04-01

**Authors:** Christopher N Balakrishnan, Robert Ekblom, Martin Völker, Helena Westerdahl, Ricardo Godinez, Holly Kotkiewicz, David W Burt, Tina Graves, Darren K Griffin, Wesley C Warren, Scott V Edwards

**Affiliations:** 1 Department of Organismic & Evolutionary Biology, Museum of Comparative Zoology, Harvard University, Cambridge, MA 02138, USA; 2 Department of Animal & Plant Sciences, University of Sheffield, Sheffield, UK; 3 Department of Population Biology and Conservation Biology, Uppsala University, Uppsala, Sweden; 4 Department of Biosciences, University of Kent, Kent, UK; 5 Department of Animal Ecology, Lund University, Lund, Sweden; 6 School of Medicine, Genome Sequencing Center, Washington University, St Louis, MO, USA; 7 Roslin Institute, Division of Genetics & Genomics, University of Edinburgh, Edinburgh, UK; 8 Current address: Institute for Genomic Biology, University of Illinois at Urbana-Champaign, 1206 West Gregory Drive, Urbana, IL, USA

## Abstract

**Background:**

Due to its high polymorphism and importance for disease resistance, the major histocompatibility complex (MHC) has been an important focus of many vertebrate genome projects. Avian MHC organization is of particular interest because the chicken *Gallus gallus*, the avian species with the best characterized MHC, possesses a highly streamlined *minimal essential *MHC, which is linked to resistance against specific pathogens. It remains unclear the extent to which this organization describes the situation in other birds and whether it represents a derived or ancestral condition. The sequencing of the zebra finch *Taeniopygia guttata *genome, in combination with targeted bacterial artificial chromosome (BAC) sequencing, has allowed us to characterize an MHC from a highly divergent and diverse avian lineage, the passerines.

**Results:**

The zebra finch MHC exhibits a complex structure and history involving gene duplication and fragmentation. The zebra finch MHC includes multiple Class I and Class II genes, some of which appear to be pseudogenes, and spans a much more extensive genomic region than the chicken MHC, as evidenced by the presence of MHC genes on each of seven BACs spanning 739 kb. Cytogenetic (FISH) evidence and the genome assembly itself place core MHC genes on as many as four chromosomes with TAP and Class I genes mapping to different chromosomes. MHC Class II regions are further characterized by high endogenous retroviral content. Lastly, we find strong evidence of selection acting on sites within passerine MHC Class I and Class II genes.

**Conclusion:**

The zebra finch MHC differs markedly from that of the chicken, the only other bird species with a complete genome sequence. The apparent lack of synteny between *TAP *and the expressed MHC Class I locus is in fact reminiscent of a pattern seen in some mammalian lineages and may represent convergent evolution. Our analyses of the zebra finch MHC suggest a complex history involving chromosomal fission, gene duplication and translocation in the history of the MHC in birds, and highlight striking differences in MHC structure and organization among avian lineages.

## Background

The major histocompatibility complex (*MHC*) is a gene-dense genomic region within which many genes play a role in vertebrate immune response. *MHC Class I *genes encode surface receptors in most nucleated cell types and facilitate immune responses to intracellular pathogens. *MHC Class II *genes also encode receptors but are restricted to antigen presenting cells of the immune system where they play a role in combating extracellular pathogens. After the binding of antigens, Class I and Class II proteins present them to CD8 and CD4 T cells, respectively. This presentation in turn triggers the adaptive immune response against the antigen. Polymorphism at *MHC *loci facilitates binding of a diversity of pathogens and this evolutionary selection pressure is thought to contribute to the high genetic variation in *MHC *loci [1]. *MHC *genes are perhaps the most thoroughly studied example of adaptive molecular evolution, representing a classic example of balancing selection [2-4]. *MHC *genes have also played an important role in studies of molecular ecology with *MHC *genotype influencing patterns of mate choice [reviewed in [5]], local adaptation [6], disease resistance [7], and the expression of sexually selected ornaments [8,9].

An *MHC *has been identified in all jawed vertebrates studied to date. A core set of genes, including *TAP, TAPBP, TNXB *and *CENP-A*, are syntenic to the *MHC *across vertebrates that have been characterized [10-12]. Therefore, the presence of these genes, along with *Class I *and *Class II *genes can be used to define the *MHC *region. In addition to *Class I *and *Class II *genes, many of the other genes in the *MHC *region also play a role in immune response. Like *Class I *and *Class II *genes, *CD1 *genes (which are MHC-linked in the chicken but not in mammals) play a role in the adaptive immune response. *CD1 *molecules present lipid, glycolipid and lipopeptides to T and NKT cells [13]. *CD1 *genes are in fact evolutionarily related to *Class I *and *Class II *genes [14-16]. Some MHC-linked genes encode proteins that interact with *MHC *molecules. *TAP1 *and *TAP2 *genes, for example, are involved in the loading of peptides onto *Class I *molecules for transport to the cell surface [17,18]. The interaction between *Class I *and *TAP *is itself mediated by *TAPBP *(tapasin).

While *MHC*s share many structural features there is also tremendous variation in their organization among species. Among birds, the chicken *Gallus gallus *has been most intensively studied, and its *MHC *(also known as *MHC-B *or B-complex) has a rather remarkable structure: a minimal essential *MHC *[19]. In contrast to humans, in which the *MHC *spans four megabases (MB) and consists of over 200 genes, the chicken *MHC *consists of only about 40 genes spanning only a few hundred kb on chromosome 16 [19,20]. In addition to the *MHC-B*, chicken *MHC Class I *and *Class II *genes are also present in a separate and unlinked cluster called the *MHC-Y *(or *rfp-Y*) region [21-25]. Even when considering both the *MHC-B *and *MHC-Y *together, the chicken MHC has fewer total genes, gene duplicates, pseudogenes, repetitive sequences, and shorter genes and introns than its mammalian counterparts.

Tight linkage of genes in the *MHC *may facilitate their coordinated coexpression [19,23,26] and coevolution in the chicken B-complex [27]. Furthermore, the suppression of recombination among *MHC *genes is thought to contribute to the evolution of gene complexes coadapted to particular pathogens and environments [26,28]. Interacting *TAP *and *Class I *genes are more closely linked in the chicken than in mammals and these genes in particular are thought to coevolve in birds [24]. Some of the strongest genotype/disease resistance correlations have been identified in the chicken [for example, [29,30]] and the simple architecture of the chicken *MHC*, with few highly expressed *MHC *genes, likely contributes to this pattern [19,23,24,26]. Due to the limited taxonomic and genomic sampling of *MHC *regions in birds, however, it remains unclear whether the streamlining of the *MHC *reflects the broader trend of reduced genome size in birds [31,32] and whether a small *MHC *represents the ancestral condition for birds. Alternatively, a small *MHC *may be a highly derived condition unique to the biology of Galliforms.

The zebra finch genome, representing the taxonomically diverse Passerine clade (approximately 5,400 species), offers the opportunity to characterize *MHC *structure in an avian lineage highly divergent from the chicken. Molecular estimates of divergence between Passerines and Galliforms indicate that they diverged between about 90 and 120 million years ago [for example, [33]]. Among birds, only two Galliform *MHC*s, the domestic chicken and Japanese quail *Coturnix japonica*, have been well characterized [34,35]. The *MHC-B *complex of another Galliform species, the turkey *Meleagris gallopavo *has also recently been sequenced and appears similar to the chicken in structure spanning about 200 kb [36,37]. *MHC *polymorphism surveys in passerines suggest that their *MHC *may differ from the structure seen in Galliforms [7,38-40]. *Class IIB *genes in particular appear to have been extensively duplicated in passerine birds, although little is known regarding the expression of these genes. Initial attempts to characterize the passerine *MHC *regions using genomic sequence data have uncovered pseudogenes and have revealed a much lower gene density than the chicken [41-43]. The number of expressed Class I genes in some songbirds also appears greater than in the chicken [44]. None of the core *MHC*-associated genes described above have been characterized in passerines making it unclear whether classical *MHC *regions have been sequenced. In this study we used the draft assembly of the zebra finch genome [45] in combination with targeted BAC sequencing, fluorescence in situ hybridization (FISH) mapping, and restriction fragment length polymorphism (RFLP) analysis to describe the fundamental features of the zebra finch *MHC*.

## Results

### Genome assembly analysis

In our scan of the zebra finch genome assembly we found one or more homologous loci for 18 of 28 investigated chicken MHC related genes (Table 1). These represent whole coding sequence (cds) or fragments of genes (one or more exons). Since several of the genes we queried had multiple loci in the zebra finch assembly, our set of sequences comprises a total of 22 manually curated *MHC *genes and eight putative pseudogenes (sequences containing frame shift mutations or premature stop codons).

**Table 1:** MHC genes identified in the survey of the zebra finch genome assembly.

*Chicken*			*Zebra Finch*			
Gene	# loci	Chr#	Locus ID	Chr#	Coordinates and orientation (+/-)	Ensembl ID
CD1	2	16	1	12	36510 -- 39728 (+)	-
			2	12	31218 -- 32904 (+)	ENSTGUG00000003538
TNXB	1	16		-	-	-
CYP21	1	16		22_random	5,200 - 8,483	ENSTGUG00000003380
CENP-A	1	16		Un	Contig 19574 (-)	ENSTGUG00000016809
C4	1	16		-	-	-
TAP1	1	16		14_random	8635 -- 8114 (-)	ENSTGUG00000015337
TAP2	1	16		-		-
Class I	2 (+MHC-Y)	16	1	22_random	954 -- 3750 (+)	ENSTGUG00000017273
			ψ L	Un	Contig 5002 (-)	-
			ψ L	Un	Contig 19247 (+)	ENSTGUG00000016646
			ψ L	Un	Contig 43472 (-)	-
			ψ L	Un	Contig 237 (+)	-
			ψ L	Un	Contig 5814 (+)	-
			ψ L	Un	Contig 29268 (-)	ENSTGUG00000014179
			ψ L	Un	Contig 24227 (-)	ENSTGUG00000015460
			ψ L	Un	Contig 19531 (-)	
			ψ L	Un	Contig 1325 (-)	ENSTGUG00000016290
			ψ C	Un	Contig 237 (+)	ENSTGUG00000015195
			ψ O	16_random	79190 -- 79374 (+)	
DMA	1	16		-	-	-
DMB	2	16		-	-	-
BRD2	1	16		Un	Contig 10922	
Class IIB	2 (+MHC-Y)	16	1	Un	Contig 1486 (-)	ENSTGUG00000013745
			1	Un	Contig 3597 (-)	ENSTGUG00000016075
			1	Un	Contig 12575 (-)	ENSTGUG00000015634
			1	Un	Contig 648 (+)	ENSTGUG00000014620
			1	Un	Contig 926 (+)	ENSTGUG00000017149
			1	Un	Contig 3052 (-)	ENSTGUG00000014503
			1	Un	Contig 11727 (+)	ENSTGUG00000015020
			2	Un	Contig 395 (+)	ENSTGUG00000016844
			2	Un	Contig 4424 (-)	ENSTGUG00000014905
			3	Un	Contig 2943 (+)	ENSTGUG00000014233
			4	Un	Contig 11297 (-)	ENSTGUG00000014649
			ψ L	Un	Contig 3510 (-)	ENSTGUG00000015846
			ψ M	22_random	279244-283106(+)	ENSTGUG00000017281
			ψ M	Un	Contig 3181 (-)	-
			ψ Q	7_random	92325-103474 (+)	ENSTGUG00000016701
			ψ R	Un	-	ENSTGUG00000017139
			ψ T	22_random	-	ENSTGUG00000017280
TAPBP	1	16		-	-	-
Blec1	1	16		Z	64162080 -- 64162784 (+)	ENSTGUG00000005208
NKr	1	16		Z	64155557 -- 64154733 (-)	-
BG	3	16		-	-	-
TRIM41	1	16		-	-	-
TRIM27	1	16		Z	64166328 -- 64164434 (-)	ENSTGUG00000005203
TRIM39	1	16		Un	Contig 15508 (+)	ENSTGUG00000014157
TRIM27.2	1	16		-	-	-
TRIM7	1	16		16_random	756 -- 1262 (+)	ENSTGUG00000015652
LAO	1	16		16_random	127,342-132,827 (+)	ENSTGUG00000016298
TRIM7.2	1	16		16_random	147414 -- 164815 (+)	ENSTGUG00000015672
KIFC1	1	16		22_random	7,798-8,481 (+)	-
Class IIA	1	16 Un		Un	Contig 28013 (+)	-
CIITA	1	14		14	6294135 -- 6303566 (+)	ENSTGUG00000004838
Ii	1	13		13	6774603 -- 6780145 (+)	ENSTGUG00000000882
B2M	1	10		10	-	ENSTGUG00000004607

Genes are listed in order according to their organisation on chicken chromosome 16. Duplicated genes are given a numbered Locus ID if they appear functional. Putative pseudogenes are marked with a ψ and given a lettered locus ID. Loci are classified based on sequence similarity such that identical sequences found in multiple places in the genome assembly are given the same Locus ID. Where Ensembl IDs have been assigned these are also given. Three genes outside of the MHC region but with a related function (CIITA, Ii, and B2M) are also included.

We found only one functional *MHC Class I *gene, which was situated on chromosome 22_random (linked to chromosome 22, but exact location and orientation unknown). In addition, there are also 10 contigs unincorporated into the genome assembly, and one contig on chromosome 16_random, that contain fragments of *Class I *genes (Table 1). Sequence differences suggest that these genes correspond to at least three different pseudogenes. In the case of *MHC Class IIB*, we found 14 contigs on chromosome Un (unmapped genomic region), and three hits on chromosomes 22_random and 7_random, containing parts of the cds. Four distinct sequences with an open reading frame spanning exons two and three appear to be functional.

The genome assembly suggests that some *MHC*-associated genes may not be as clustered in the zebra finch *MHC *as they are in the chicken. *Blec1*, *NKR *and *TRIM27*, for example, map to the Z chromosome in the zebra finch genome assembly, while two *CD1 *loci map to chromosome 12. Many of the other genes for which we searched, however, mapped to chromosome UN or were not found in the assembly (Table 1).

### BAC screening, sequencing and gene prediction

**Figure 1:**
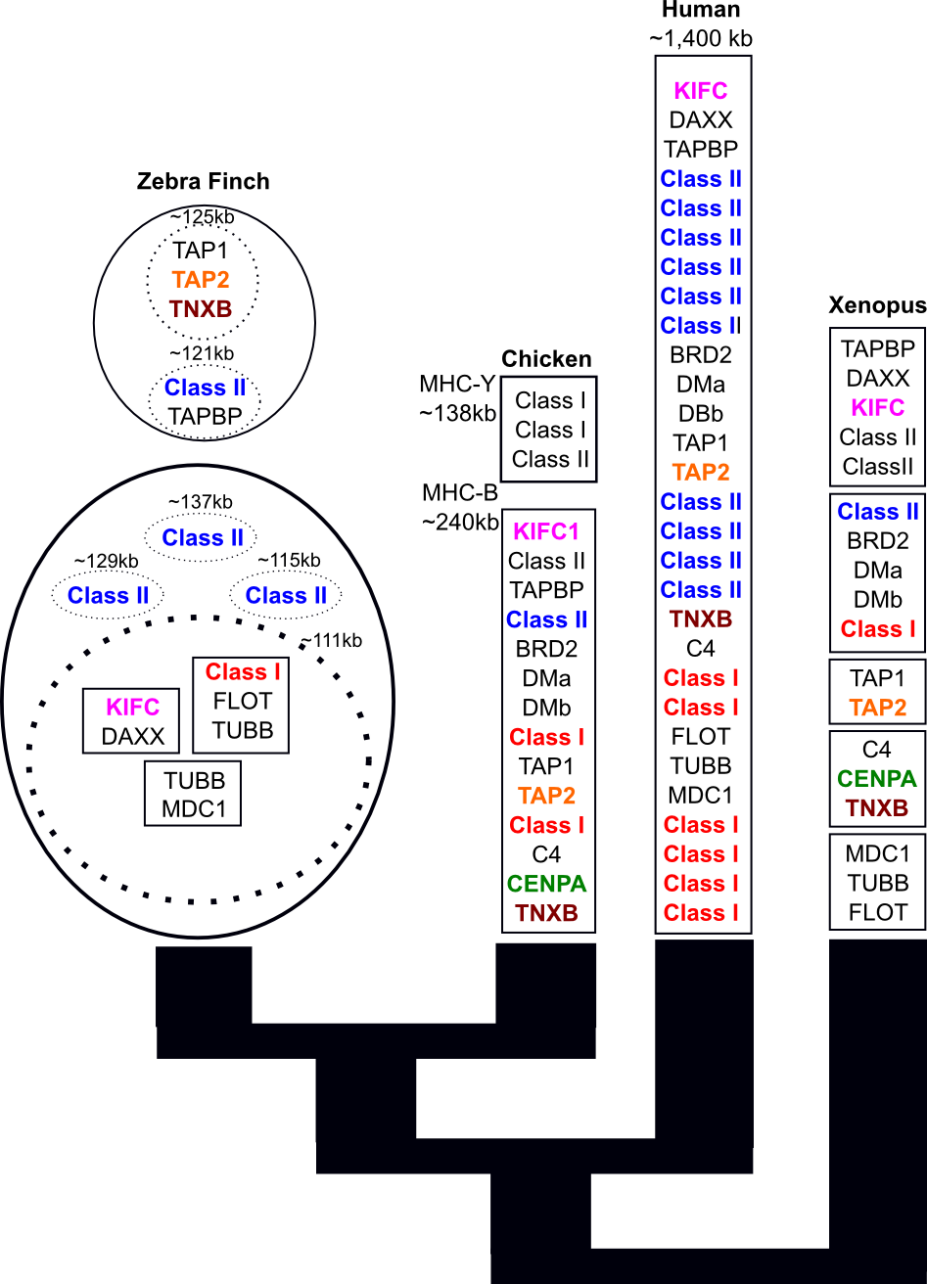
**Schematic diagram highlighting results of BAC clone assembly and annotation, FISH mapping, and evolutionary comparisons**. For zebra finch, genes within boxes are linked in a single BAC contig. Contigs within dashed ovals are linked by known location within a single BAC but the order is uncertain. BACs that map to the same chromosome via FISH mapping in are within a solid oval (see also Figure 4 for FISH mapping results). For chicken boxes represent *MHC-B *and *MHC-Y *regions. For *Xenopus *boxes represent sequenced BACs whose chromosomal organization is unknown. For clarity, not all genes of the *MHC *are shown.

**Figure 2:**
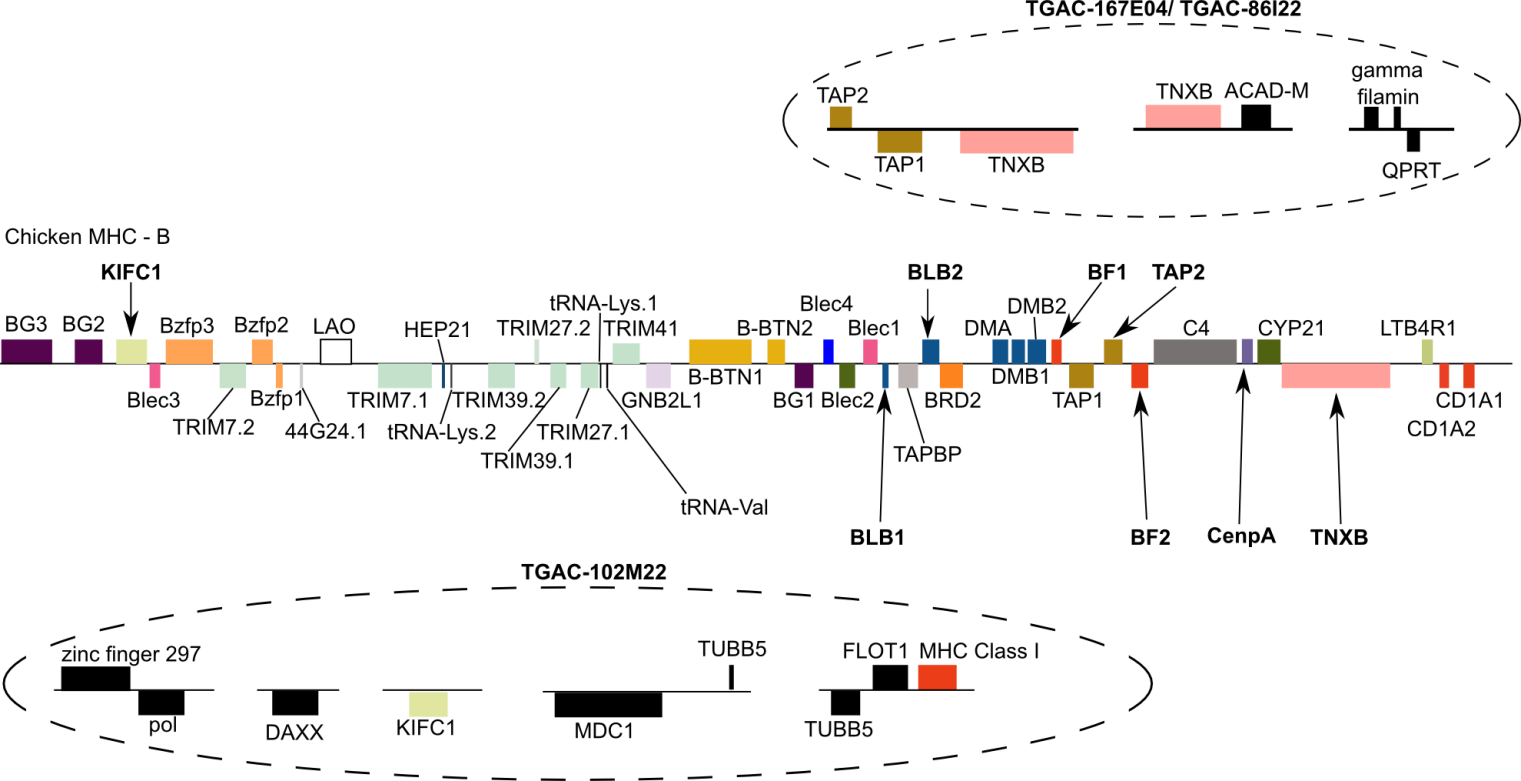
**Genomic map of the Chicken MHC - B complex after Shiina et al**. [20]** compared to two sequence zebra finch *Class I *clones**. While *KIFC *and *MHC Class I *were identified in a single BAC, no orthologs of the intervening chicken genes were found in zebra finch. An *MHC Class I *gene was not found in the *TAP *containing zebra finch clone despite the proximity of these genes in the chicken *MHC*. Following the chicken naming scheme, class I *MHC *genes in chicken are denoted *BF1 *and *BF1*, and *class IIB *genes are denoted *BLB1 *and *BLB2*. Genes targeted in the BAC screening are marked with arrows.

We further characterized the zebra finch *MHC *by isolating and sequencing *MHC*-containing BAC clones. We first identified 96 clones that hybridized strongly with a probe targeted to exon 3 of an *MHC Class IIB *gene. Four of these BACs were selected for sequencing (hereafter *Class II *clones). Because of the large number of *Class IIB *positive clones, we conducted further screening using overgo probes targeted to five conserved genes linked to the *MHC *across a diversity of taxa (Table 2; Additional File 1). For this second screening, we screened a different BAC library derived from the same zebra finch individual as the whole genome sequence (see methods). Positive clones were found for each of the five genes: *MHC Class I *(n = 21), *KIFC *(n = 56), *CENP-A *(n = 44), *TAP2 *(n = 14), and *TNXB *(n = 11). Probes for three pairs of genes were found to cohybridize to individual BAC clones: *MHC Class I *and *KIFC*, *MHC Class I *and *TNXB*, and *TNXB *and *TAP *(Table 2). One BAC clone containing each of these three gene pairs was chosen for 6× sequencing (hereafter *Class I clones*). No clones were positive for both *TAP2 *and *MHC Class I*, suggesting that these two genes are not closely linked in the zebra finch as they are in the chicken. *CENP-A *probes also did not cohybridize with any of the other MHC genes, again indicating a lack of close linkage observed in other species (Figures 1 and 2). We did not sequence any of the *CENP-A *positive clones.

**Table 2:** Results of overgo hybridization of zebra finch BAC library.

	KIFC	Class I	TNXB	TAP2	CENP-A
Positive Clones	56	28	11	14	44
				
Co-hybridizing clones	16				
				
		1			
				
			4		

Cohybridization patterns link all genes but *CENP-A*. The clone that was positive for *TNXB *and *Class I *appears to contain a Class I pseudogene.

Each clone was assembled into multiple ordered contigs (Table 3). The fragmented nature of the BAC assemblies is expected given the coverage, but was exacerbated by high repeat content (see below; Additional File 2). Sequence analysis of two *Class I *clones, *TGAC-86I22 *and *TGAC-167E04*, revealed extensive sequence overlap and thus were assembled together (Table 3). To improve the assembly for the *Class II *clones we generated additional sequencing reads. Because the *Class I *clones were derived from the same zebra finch as the whole genome sequence, we were also able to incorporate sequence reads from the whole genome sequencing effort to improve the assembly of these BACs.

**Table 3:** Description of BAC assemblies.

BAC Clone	GenBank #	# contigs	largest contig	average length	total length
TGAA-157B03	AC192433	2	79,546	60,262	120,523
TGAA-323J16	AC191651	4	75,043	32,336	129,342
TGAA-351E14	AC191861	3	91,199	45,792	137,376
TGAA-047O03	AC192431	3	58,397	38,629	115,189
TGAC-102M22	AC232985	12	20,620	9,274	111,298
TGAC-167E04/TGAC-86I22	AC232854	17	25,067	7,355	125,027
					
				total assembled length:	738,755

Most of the BAC assemblies contained at least a fragment of the genes expected based on the probes used to identify them (Additional File 3). Class I clones contained a number of genes of interest. The assembly of clones TGAC-86I22 and TGAC-167E04 contained sequences with similarity to *TAP1*, *TAP2*, and *TNXB *genes, and clone TGAC-102M22 contained *KIFC*, *DAXX*, *TUBB*, *Class I*, and *FLOT *(Figure 1). Although numerous genes separate *KIFC *and *Class I *genes in chicken (Figure 2) we did not find evidence for these genes within this BAC. *DAXX*, *TUBB *and *FLOT *have not yet been identified in the chicken *MHC *but are associated with the *MHC *in other vertebrates (Figure 1). Because of gaps in the BAC assemblies complete coding sequences could not always be reconstructed (for example, *TAP2*, Additional File 3). Although TGAC-86I22 hybridized with both *Class I *and *TNXB *probes, sequencing only revealed a small region with similarity to the *Class I *3' UTR in the great reed warbler (e-value: 8e-19, identities: 166/230; 72%). Polymerase chain reaction (PCR) screening of this clone also identified a stretch of a short exon 3 sequence (200 bp) that is identical to the expressed locus. The UTR region, however, is distinctive in sequence from the expressed zebra finch *Class I *3' UTR and BLAST searches of brain expressed sequence tags (ESTs) and 454 sequencing data from multiple tissues suggest that this locus is not expressed [46]. Together this suggests that clone *TGAC-86I22 *contains an *MHC Class I *pseudogene.

**Figure 3:**
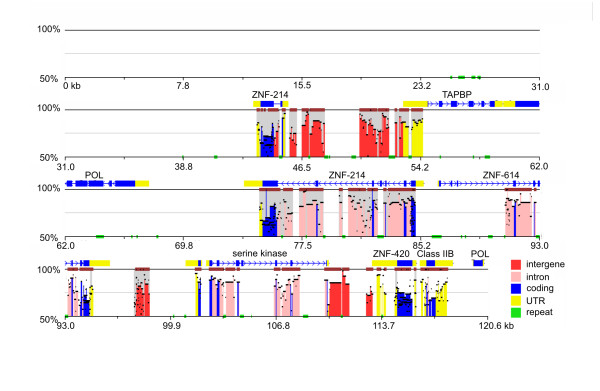
**Sequence conservation and alignment diagram using Zpicture**. Zebra finch BAC 157*B*03 and previously sequenced cosmid clone (rwcos3) from red-winged blackbird [48] were compared highlighting regions of sequence conservation. The Y axis in each panel represents the percent similarity. Exons (blue boxes), UTRs (yellow boxes) and intergenic regions are based on FGENESH predictions, and repeats (green boxes) are predicted by Zpicture [47] (using Repeatmasker). Regions of sequence similarity (brown boxes) not only include the Class IIB gene, but also the zinc finger-like sequences identified. Gene names are based on best BLAST hits. The ordering of genes is based on the zebra finch BAC assembly and is not necessarily the same in the red-winged blackbird.

*Class II *clones contained numerous predicted genes with sequence similarity to zinc finger genes, as well as *gag *and *pol *proteins (endogenous retroviral genes). Aside from these and the expected Class IIB sequences, however, only one other gene of interest was found. A gene whose best blast hit matched the first four exons of the turkey *TAPBP *gene (blastx e-value 6e-22) was found in clone *TGAA-157B03*. Sequence conservation mapping using Zpicture [47] of this clone and a previously sequence red winged blackbird *Agelaius phoenicius *Class II region [48], highlight sequence similarities in the coding and UTRs of predicted genes, as well as in some putative intergenic regions (Figure 3).

### FISH mapping of BAC clones to zebra finch chromosomes

**Figure 4:**
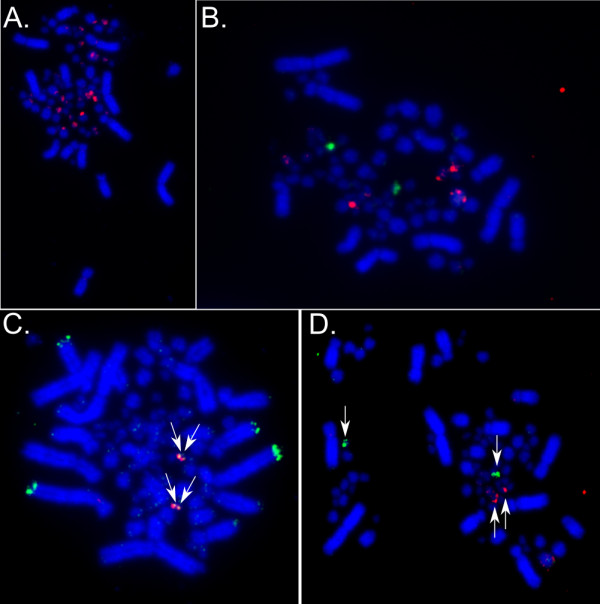
**FISH mapping of BAC clones**. **A) **Single color FISH mapping of TGAC-157B03 reveals extensive cross-hybridization across chromosomes. Similar results were observed for other Class II clones presumably as a result of their high repeat content. **B) **Lack of cohybridization between Clones TGAC-102M22 and a known chromosome 22 BAC indicates that TGAC-102M22 is not on chromosome 22 as indicated by the genome assembly. **C) **Dual color FISH of TGAC-86I22 (red) and TGAC-167E04 (green) indicating cohybridization of these clones, a result also supported by sequence analysis. These clones were assembled together, and contain g-filamin, TNXB, TAP1 and TAP2 genes. **D) **Clones TGAC-102M22 (red) (contains MHC Class I, FLOT, TUBB, KIFC and DAXX) and TGAC-86I22 map to different chromosomes. Key components of the classical MHC therefore map to different chromosomes in the zebra finch genome.

Single-color FISH mapping experiments revealed that sequenced *Class II *BACs (*TGAA-157B03, TGAA-351E14, TGAA-323J16 *and *TGAA-47O03*) hybridized to several pairs of microchromosomes each (Figure 4), likely due to the high repeat content in these clones (see below). In contrast, sequenced *Class I *BACs (*TGAC-86I22, TGAC-102M22 *and *TGAC-167E04*) each hybridized to one pair of small microchromosomes. Some BACs also cross-hybridized to repeats in the centromeric and telomeric regions of macrochromosomes (for example, Figure 4). Linkage analysis by dual color FISH demonstrated that BACs *TGAC-102M22 *(containing presumptive *Class I*, *FLOT, TUBB, KIFC, DAXX*), and three *Class II *BACs (*TGAC-323J16, TGAC-351E14 *and *TGAC-47O03*) shared hybridization to one pair of small microchromosomes. Sequenced BACs *TGAC-86I22 *and *TGAC-167E04 *(containing presumptive *TNXB, TAP1, TAP2*) and *157B03 *(*Class II*, *TAPBP*), however, hybridized to a different pair of small microchromosomes (Figure 4). *MHC *genes are thus found in two linkage groups on separate chromosomes in zebra finch.

To further test whether *TAP *and *MHC Class I *genes are syntenic, we conducted five additional two-color FISH experiments with BAC clones that were positive for *TAP2 *and *MHC Class I*. While some *MHC Class I *probes hybridized to multiple microchromosomes, in only one case did we find colocalisation of *Class I *and *TAP2 *probes (Table 4, Additional File 4). In this case, *MHC Class I *probes hybridized to multiple microchromosomes, and the colocalisation occurred on the W sex chromosome. It is therefore likely that this colocalisation is due to nonspecific binding, and the repetitive nature of the avian W chromosome. In total we have four cases in which *TAP2 *probes hybridize unambiguously to a single microchromosome and in all of these, *Class I *maps to a different chromosome.

**Table 4:** Two-color FISH mapping results of putative TAP 2 and MHC Class I-containing clones.

MHC Class I	Mapping	TAP 2	Mapping	Colocalisation
TGAC-102M22*	1 micro	TGAC-167E04*	1 micro	No
TGAC-102M22*	1 micro	TGAC-86I22*	1 micro	No
TGAC-15A11	1 micro	TGAC-95I13	centromere macro	No
TGAC-181L18	1 micro	TGAC-53B12	Z	No
TGAC-12A09	micros, W	TGAC-14G17	1 micro and W	Yes
TGAC-250C06	micros	TGAC-139M05	1 micro	No
TGAC-252P06	1 micro	TGAC-249G24	1 micro	No

* Clones sequenced in this study

Most clones mapped to a single microchromosome (1 micro) where as some showed non specific binding to multiple microchromosomes (micros). A few clones mapped to sex chromosomes (Z and W), and one mapped to the centromeric region of a macrochromosome (macro). In only one cases did *TAP2 *and *Class I *clones colocalise, and that was on the W chromosome.

In order to identify the zebra finch chromosomes corresponding to the two zebra finch linkage groups, we performed dual-color FISH experiments in which one BAC from one of the two linkage groups (*TGAC-102M22 *or *TGAC-86I22*) was co-hybridized with a non-MHC BAC with known chromosomal location (Additional File 5). These experiments covered all microchromosomes for which BACs are currently available (chromosomes 9 to 15 and 17 to 28). Neither of the two *MHC *linkage groups mapped to these chromosomes, suggesting that both microchromosomes to which the *MHC *BACs mapped may indeed correspond to parts of zebra finch chromosome 16, the only chromosome for which we do not have known BACs.

### Polymorphism survey via RFLP/Southern Blot

**Figure 5:**
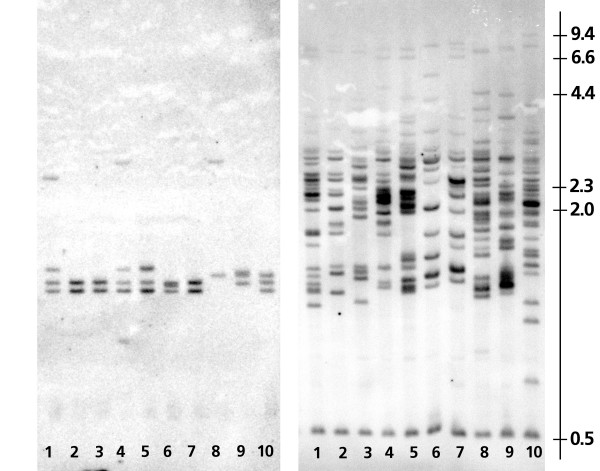
**RFLP/Southern Blot of 10 captive zebra finches**. Individuals 1 to 7 are from a captive American population and individuals 8 to 10 are from a Swedish population. The left panel shows the banding patterns using a Class I probe and the right panel shows the results using a Class II probe. Results from Class I analysis suggest a minimum of two loci whereas Class II probes indicate a very large lumber of loci (mean number of bands = 19 +/- 4.6, range: 12 to 27).

To test our findings on the numbers of *Class I *and *Class IIB *genes, and as a preliminary survey of gene number and intraspecific polymorphism, we conducted a RFLP analysis using probes targeted to these loci. There are clearly a larger number of RFLP bands for *MHC Class IIB *(range = 12 to 27) than for Class I (range = 2 to 4) and this also suggests that there are more *Class IIB *genes than Class I genes in zebra finches (Figure 5). This difference in gene number is not likely due to differences in the sequence similarity of probe and target for *Class I *and *IIB *probes as we would expect that the longer *Class I *probe (280 bp) should hybridize to a larger number of fragments than the *Class IIB *probe (207 bp). We repeated this hybridization twice using different *Class I *and *II *probes (data not shown). For *MHC Class I *there are two to four RFLP fragments in the captive zebra finches from the US (ind 1 to 7) and two to three fragments in the zebra finches from Sweden (ind 8 to10). For *MHC Class IIB *there are 12 to 20 RFLP fragments in the zebra finches from the US and as many as 27 fragments in the three zebra finches from Sweden.

### Comparative analysis of MHC genes

**Figure 6:**
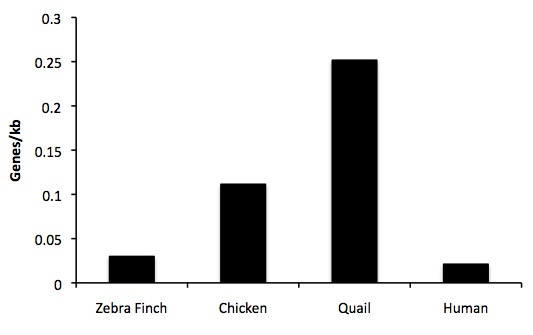
**Comparison of gene density across three avian lineages and the human HLA region**. Estimates from zebra finch are based on two BAC assemblies (TGAC-102M22 and TGAC-167E04/TGAC-86I22) containing 11 predicted genes.

**Figure 7:**
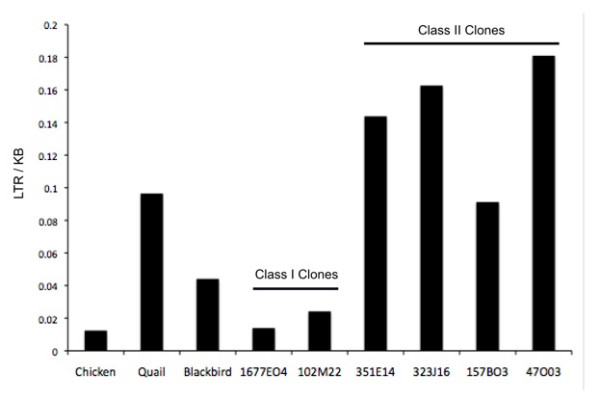
**Long terminal repeat (LTR) content in avian MHC regions**. Chicken (AB268588), Quail (AB078884.1), and Blackbird (AF328738) sequences from Genbank are compared with sequenced zebra finch BACs.

In order to explore the evolution of the compact avian *MHC *structure observed in chicken, we estimated the average gene density in quail, chicken, zebra finch and human. Interestingly, the estimated gene density in the zebra finch is similar to that in humans, and distinctive from both quail and chicken (Figure 6). Repeat content also differs markedly between the chicken *MHC *region and the zebra finch BACs. Long interspersed nuclear elements (LINEs) occur at frequency of 0.07 per kb in chicken versus 0.02 per kb across the 739 kb of zebra finch BAC sequence. Long terminal repeat (LTR) content, specifically in the form of *ERV1 *elements, was exceptionally high in zebra finch *Class II *sequences, occurring at frequency of 0.14 per kb, whereas the chicken MHC is depauperate in LTR at 0.01 per kb (Figure 7). Zebra finch *Class I *clones sequenced here more closely resemble the chicken content, but still had higher LTR content (0.02 LTR/kb).

**Figure 8:**
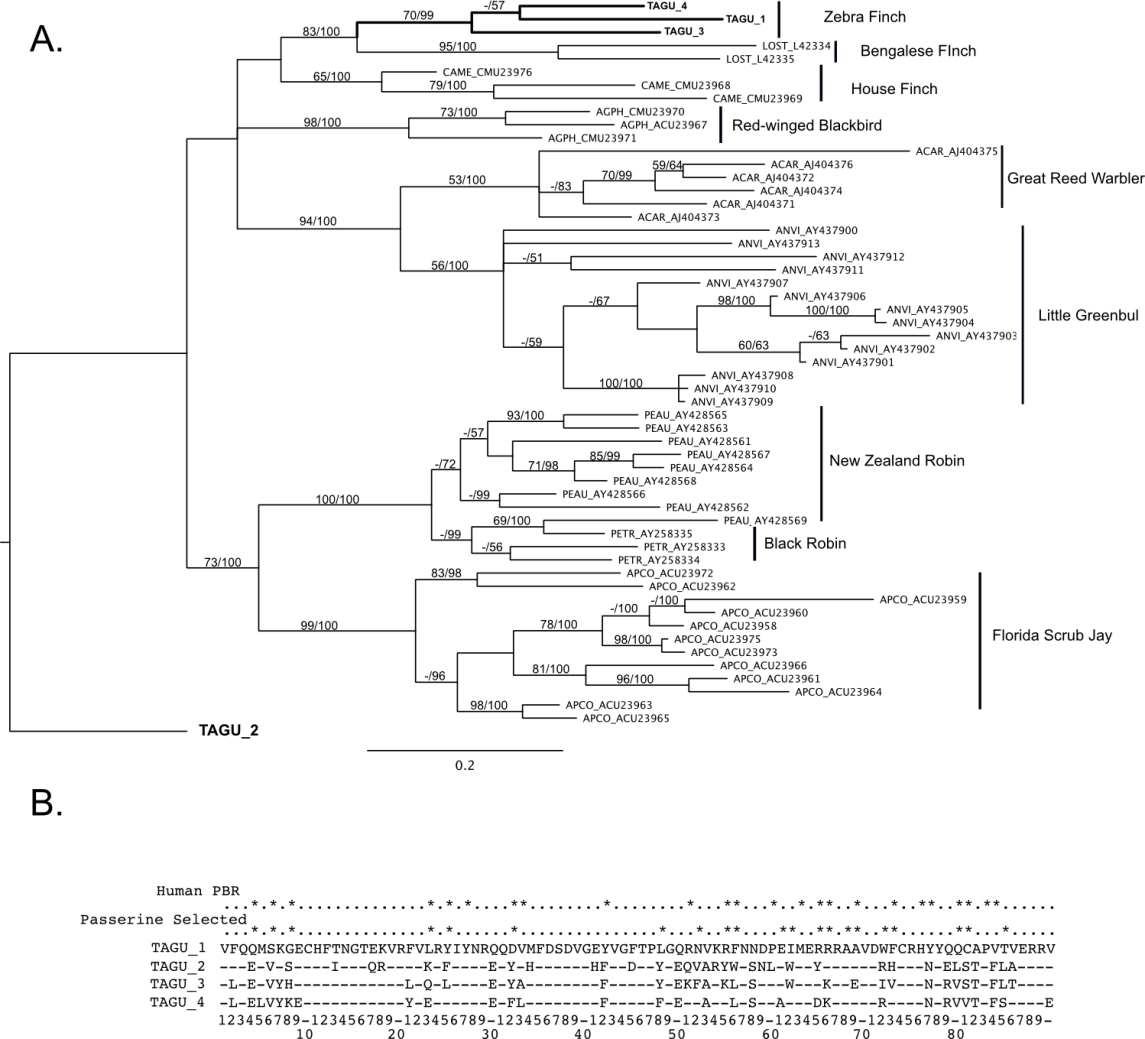
**Phylogenetic analysis and selection on MHC Class II sequences**. **A) **Phylogenetic relationships among passerine MHC Class II exon 2 and 3 sequences. Four sequences with open reading frames were found in the zebra finch genome. The remaining sequences are from GenBank. The root of the tree was placed at a divergent zebra finch lineage (TAGU 2) based on a larger analysis in which non-passerine sequences were included. TAGU 1 to 4 correspond to loci 1 to 4 in Table 1. **B) **Predicted amino acid sequences of the second exon of four apparently functional zebra finch MHC Class IIB genes. Stars represent sites showing evidence of selection in passerine birds. Note the correspondence between sites showing evidence of selection in passerines and the predicted peptide binding region in humans.

To examine the evolutionary relationships among *MHC *genes, we placed exons 2 and 3 of four putatively functional zebra finch *MHC Class IIB *sequences in a phylogenetic context by comparing them with other passerine sequences from GenBank. Exon 2 of the *Class IIB *gene encodes the protein that forms the peptide binding region of the *Class II *protein, and exons 2 and 3 have been amplified and sequenced in a diversity of bird taxa. Bayesian phylogenetic analyses using both exons concatenated together grouped three zebra finch sequences in a highly supported clade (Figure 8). Using outgroups to the passerine sequences (data not shown), a divergent zebra finch locus was placed basal to all passerine sequences, between passerine and non-passerine sequences. Thus for analyses of passerine sequences we rooted the tree at this zebra finch sequence (Figure 8). We also analyzed exons 2 and 3 separately. These results reflect previously described differences among the exons [for example, [38]] so are not described further here. Phylogenetic analyses of exon 3 sequences from *MHC Class I *also placed zebra finch *Class I *sequences in a strongly supported clade (Additional File 6).

Passerine sequences were analyzed using Phylogenetic Analysis using Maximum Likelihood (PAML) [49,50], to test for the influence of positive selection on *MHC Class I *exon 3, and Class IIB exon 2. Two pairs of models were compared in CODEML, M1 (nearly neutral) versus M2 (positive selection) and M7 (β) versus M8 (β and ω > 1) [49,50]. For Class I exons both tests indicate that models incorporating positive selection provide a better fit to the data than do neutral models (M1 vs. M2, 2ΔlnL = 270.5, *P *= 0.00; M7 vs. M8, 2 ΔlnL = 271.7, *P *= 0.00). Bayes Empirical Bayes analyses [49] identified 10 sites with ω > 1 (posterior probability > 0.95; see Additional File 6). Analyses of MHC Class IIB exon 2 also suggest a strong influence of positive selection (M1 vs M2, 2 ΔlnL = 540.4, *P *= 0.00; M7 vs M8, 2ΔlnL = 511.0, *P *= 0.00) with 21 sites with ω > 1 (Figure 8). In both Class I and Class IIB, these sites correspond well with the peptide binding region (PBR) from the human MHC [51,52] and with selected sites identified in birds of prey [53,54].

## Discussion

We have provided here a detailed characterization of the zebra finch *MHC*. There is clear cytogenetic evidence that *MHC *genes map to at least two different chromosome pairs in the zebra finch. If the chicken *MHC *represents the ancestral state, the situation in the zebra finch may have arisen through fission of chromosome 16 or a translocation of part of it to another pair of microchromosomes. The hypothesis of chromosomal fission is consistent with the finding that the *MHC *BACs did not map to zebra finch chromosomes 9 to 15 or 17 to 28, and that the microchromosomes recognized by these probes were small.

The finding of *MHC *genes on two chromosomes in the zebra finch is particularly intriguing because *TAP *genes map to one of them, whereas an expressed *Class I *gene (and a number of other *MHC*-associated genes) maps to a distinct chromosome (Figure 1). This finding is unexpected because *TAP *and *Class I *genes functionally interact and are syntenic in most MHCs studied to date including both chicken and humans [reviewed in [10], but see [55,56]]. In chicken this tight linkage is thought to result in coevolution between *TAP *and *Class I *genes and strong correlations between *MHC *haplotype and disease resistance [reviewed in [57]]. *TAP *genes in mammals, while generally syntenic, are not as closely linked to *Class I *as they are in Galliform birds. The separation of *TAP *and *Class I *in mammals has been hypothesized to have resulted in their evolutionary independence and in turn led to high levels of duplication and divergence in *Class I *genes [56]. This dissociation is perhaps most clearly illustrated by the tammar wallaby *Macropus eugenii *in which *Class I *sequences have been found dispersed across seven chromosomes [56]. The separation of *TAP *and *Class I *genes in the zebra finch may therefore represent convergent dissociation of these genes.

An alternative explanation for the separation of *Class I *and *TAP *genes in the zebra finch is that the regions sequenced here could represent duplication blocks. The sequenced Class I locus could even be related to the *MHC-Y *region of chicken. Phylogenetic analyses of zebra finch *Class I *and chicken *Class I *(*MHC-B *and *MHC-Y*), however, suggest that our sequenced Class I gene is not the ortholog of a chicken *MHC-Y *gene as chicken (including *MHC-Y*) and zebra finch sequences are reciprocally monophyletic (Additional file 6). It is also possible that a second Class I gene resides on the same chromosome as *TAP1 *and *TAP2 *and therefore, that Class I and *TAP *are actually syntenic. In fact, a sequenced BAC was positive for both *MHC Class I *and *TNXB*; Another four clones were positive for *TNXB *and *TAP2 *suggesting a possible linkage between these *MHC Class I *and *TAP2*. Based on a divergent sequence and a lack of expression, we suggest that this *Class I *sequence is a pseudogene. Even if it were not a pseudogene, *TAP *and this *Class I *gene would be much more distantly located in zebra finch than they are in chicken and would be free of the linkage seen in the chicken. The whole genome assembly, digital expression profiling [46] and EST data suggest only one full-length, expressed, *Class I *gene. It is also possible that there is a second set of *TAP *genes that we have not sequenced. Given the extremely low coverage of TAP genes in the genome trace archives (for example, only one read covering *TAP2*), it is unlikely that TAP genes have been duplicated. FISH mapping of five pairs of putative *TAP2 *and *MHC Class I *clones further supports the lack of synteny among *TAP *and *Class I *genes (Table 4). Together these findings suggest that the *Class I *and *TAP *are not linked in the zebra finch. In addition to *Class I *loci identified in the BACs, we identified three distinct *Class I *sequences that appear to be pseudogenes. One of the putative pseudogenes only contains exon 2, one only contain exons 4 to 6, while the third contains exons 1 to 3. Because the probes used in RFLP analyses target exon 3 only one of these pseudogenes would be reflected in the RFLP banding patterns. While the zebra finch appears to possess only one expressed *Class I *locus, the great reed warbler *Acrocephalus arundinaceous*, another passerine species, expresses multiple *Class I *loci [44]. An intriguing possibility is that the dissociation of *TAP *and *Class I *in ancestral passerines preceded the radiation of *Class I *genes in some passerine groups [44] as has been suggested for the wallaby [55,56].

*Class IIB *genes in zebra finch are highly duplicated as evidenced by the genome assembly, BAC sequencing and the RFLP analysis. We identified 10 distinct *Class IIB *sequences in the genome assembly (Table 1) some of which appear to be pseudogenes. These findings corroborate previous surveys of *Class IIB *variation in other passerine birds [40,58,59]. Another feature of zebra finch *Class IIB *regions is their high LTR content, mostly in the form of ERV elements (Figure 7). The finding of multiple zinc-finger genes and retroelements in proximity to *Class II *genes was also presaged by multikilobase *MHC *sequences from red-winged blackbirds, which showed a similar pattern [43,48]. Given the large number of *Class IIB *duplicates and pseudogenes we speculate that duplication may have been related to the presence of retroviral sequences. Thus, the passerine *MHC Class IIB *may have been invaded by endogenous retroviruses much like the primate *Class I *[28]. Endogenous retroviruses have also been implicated in the duplication of wallaby *Class I *genes and their spread across multiple chromosomes [56].

Given the FISH mapping results and the whole genome assembly, *MHC *genes appear to be located on even more than two chromosomes. The genome assembly suggests that homologs of chicken *MHC *genes have been dispersed in the genome. There are at least three possible explanations for this: 1) There have been chromosome rearrangements for these genes between the chicken and zebra finch; 2) The contigs containing these genes have been misplaced in either the chicken or the zebra finch genome assembly; 3) The zebra finch gene identified is not the true ortholog of the chicken gene. Chicken *MHC *genes placed on different chromosomes in the zebra finch assembly compared to the chicken include *MHC Class I *(*Chr22_random*), *CD1 *and *CD2 *(*Chr12*), and *NKR, Blec1 *and *TRIM27 *(*ChrZ*) (Table 1). *The MHC Class I *gene placed on chromosome 22 and its surrounding region in the assembly is essentially identical to that in our sequenced BAC. This sequenced BAC did not cohybridize with two known chromosome 22 BACs (Figure 4B; Additional file 5), so the placement of this Class I region on chromosome 22 appears to be an assembly artifact. Rather, the FISH mapping results suggest that these genes are in fact on chromosome 16 as they are in chicken. The genome assembly data underlying the placement of CD1 genes on chromosome 12 is also somewhat uncertain, with no BAC-end sequences linking contigs containing these genes to chromosome 12. Further work will be needed to test whether the genome assembly has properly placed these genes. Contigs containing, *Blec1, NKr *and *TRIM27*, however, are linked by BAC-end sequence pairs to the Z chromosome, making it likely that these are appropriately placed in the assembly.

A number of core MHC-associated genes including *DMA, BG, C4, TNXB, TAP2 *and *TAPBP *are conspicuous by their absence in the zebra finch genome assembly (Table 1). There is no reason, however, to believe that these are truly absent in the zebra finch as they are present in a wide range of other vertebrates and are crucial for MHC function. More likely, these genes cannot be identified due to the incomplete assembly of zebra finch chromosome 16. *TAP2, TAPBP *and *TNXB*-like sequences, for example, were found in the BAC sequences but are not represented in the genome assembly. Many of the zebra finch MHC-related genes identified in the genome scan map to linkage groups in chromosome unknown. This again appears to be a result of the incomplete assembly of chromosome 16. The problem of assembling chromosome 16 is likely due in part to the highly duplicated MHC region in combination with the high repeat content in these regions.

BAC sequencing revealed two genes, *FLOT *and *DAXX*, that are MHC-linked in non-avian vertebrates [10,11], but have not been described in chicken. The relatively close linkage to MHC Class I and II genes of *FLOT, TUBB *and *DAXX *in the zebra finch is actually more similar to the organisation in some teleost MHCs [for example, [12]] than it is to either *Xenopus *or the human MHC, where *DAXX *is physically distant from the *FLOT *and *TUBB *genes. Chicken chromosome 16, like the zebra finch, is not well assembled at this point so it is possible that these genes will be found as the chicken assembly continues to improve.

Phylogenetic analyses highlight the clustering of Class IIB loci by species rather than by orthology relationships, suggesting a history of concerted evolution, at least on portions of the genes [38,60,61]. We did, however, identify a unique Class IIB lineage that falls at the base of all other passerine Class II sequences. This appears to be a novel locus that has not previously been sequenced in birds and it is unknown whether it is expressed and/or polymorphic. Further analysis will be needed to clarify the role of this locus but its discovery underscores the utility of genomic approaches (rather than PCR amplification using degenerate primers) for characterizing MHC genes in birds. Tests of selection using zebra finch and other passerine MHC sequences support a strong role of selection in shaping patterns of polymorphism in the peptide binding region of Class I and Class II genes in passerines. The specific sites under positive selection are similar to those previously identified for other bird groups [53,54] and they closely match the peptide binding regions in humans [51,52]. High variability among individuals in RFLP banding patterns support the prediction that MHC Class IIB genes are influenced by balancing selection.

Among birds, there is tremendous variation among lineages in the number of MHC genes. In quail [34], red-winged blackbird [42,48] and the zebra finch, there are multiple Class II genes. Most non-passerine species, in contrast, appear to have only between one and three loci [60,62,63]. Given the derived phylogenetic position of passerines [64], these patterns imply that in terms of Class II genes, a minimal MHC may be ancestral for birds [60,62]. Because of the extensive variation among avian lineages in the number of Class I genes [for example, [34,44,65]], it remains unclear what the ancestral condition for Class I genes might be.

## Conclusions

We have made significant progress towards the understanding of the complex structure of the zebra finch MHC, the first such analysis from a representative of the diverse passerine radiation. Although the genome assembly and BAC sequencing are fragmentary, the zebra finch appears to possess an MHC differing markedly from previously described avian MHCs. The genomic architecture of the zebra finch MHC highlights the dynamic nature of MHC evolution. The evidence for gene duplication, pseudogenization and the distribution of MHC genes on multiple chromosomes in the zebra finch are particularly striking when measured against the compact MHC of the chicken present on a single chicken microchromosome. Further genomic characterization of MHCs from a broader diversity of birds, as well as further refinement of the zebra finch MHC assembly, will continue to refine our picture of MHC evolution in birds.

## Methods

### Genome assembly scan

We searched the zebra finch genome assembly extensively for MHC genes using a variety of methods. Chicken MHC genes and proteins were downloaded from the National Center for Biotechnology Information (NCBI) website and blasted (blastn and tblastn) against the published version of the zebra finch genome and the available EST library and MHC containing BACs (see below). Since many of the genes of the MHC are diverging quickly we used rather relaxed blast settings (high minimum e-value and low w). For especially tricky multigene families and genes not found using the regular blast searches we constructed alignments using several vertebrate species and searched using conserved regions only. We also constructed a hidden Markov model of conserved features using the program HMMER 2.3.2 [66] and used the output consensus sequence in an additional blast search. The HMMER model was also used with the program Wise2 [67] in an additional attempt to identify corresponding exons in the zebra finch genome.

Regions in the zebra finch genome with significant hits on one or more chicken MHC exons were aligned to each of the chicken exons from the target gene using ClustalW [68] and checked manually in BioEdit [69]. Zebra finch sequences matching chicken MHC exons were extracted and complete or partial coding sequences of genes were blasted (blastx) back against the chicken RefSeq protein database. Hits with a best reciprocal blast with an e-value of less than 1e-05 against the target gene in chicken were considered to be orthologs.

Most of the genes were also identified using automated annotation of the zebra finch genome. In these cases we have included the accession numbers for the ENSEMBL entries (Table 1). These results, however, were not available to us at the time we conducted our analysis and have not affected our gene finding. Instead our manual annotation provides support for many of the genes identified using the computerised ENSEMBL annotation [70]. Also note that in some cases there are slight differences between the sequences presented here and the sequences with the provided ENSEMBL IDs.

### BAC screening, sequencing and gene prediction

We characterized the zebra finch MHC by isolating and sequencing MHC-containing BAC clones. To generate a probe for MHC ClassIIB we PCR amplified exon 3 using degenerate primers described by Edwards et al. [71]. Probes were then radioactively labeled and hybridized with eight BAC filters following previously described protocols [72]. BAC filters were purchased from the Arizona Genomics Institute http://www.genome.arizona.edu. Clones from the AGI library are listed by names with the *TGAA *prefix. Positive clones were fingerprinted and four clones representing two pairs of putatively overlapping clones were sequenced to 6× coverage using an Applied Biosystems 3730 sequencer (Foster City, CA, USA). Because MHC Class IIB clones had apparently high repeat content, additional plasmid end reads were generated to improve the assembly.

We conducted additional screening using oligonucleotide probes targeted to five conserved genes linked to the MHC across a diversity of taxa (Table 2). This second round of screening was conducted using a different BAC library (TGAC), available through the Clemson University Genome Institute because this library was generated using DNA from the same individual zebra finch as the genome itself. Screening was done using previously established protocols [73]. Oligos were typically 24-mers (Supplemetary Table 1) overlapping by 8 bp to generate a radiolabeled double-stranded 40-mer. These 40-mers were then pooled by gene and hybridized against the filters to identify BACs containing the specific gene sequence. Once the clones were identified, they were fingerprinted and end sequenced to confirm their location in the region. For these BACs we incorporated overlapping reads from the genome into the final BAC assemblies to increase contig length and improve ordering. Individual BAC assemblies were created with PHRAP [74] and assessed for contiguity. BAC assemblies were then manually examined for misassemblies and if they were found, the data was sorted as best as possible by using forward and reverse pair data. Consensus sequence blocks for each clone were then ordered and subjected to further analysis.

We used FGENESH [75] and GENSCAN [76] to predict genes contained within the BAC sequences. FGENESH uses a hidden Markov model (HMM) for gene prediction, and we used both human and chicken databases for gene prediction. Predicted amino acid sequences were blasted (blastp) against the non-redundant protein database in Genbank. Predicted genes with strong blast hits were given putative gene names, and were visually inspected to further confirm orthology with known genes. We assessed repeat content of clones using RepeatMasker [77] and chicken repeat libraries.

### FISH mapping

Zebra finch chromosome preparations were made as previously described [78]. BACs were isolated using the Qiagen Plasmid Midi Kit (Crawley, UK). A total of 500 ng of isolated BAC DNA were labelled with biotin-16-dUTP or digoxigenin-11-dUTP (Roche Applied Science, Burgess Hill, UK) by nick translation and resuspended in 10 μl of hybridization buffer (50% formamide, 20% dextrane sulphate in 2×SSC). Slides with metaphase chromosomes were dehydrated in an ethanol series (70%, 80%, 100%, three minutes each), aged for one hour at 75°C and treated with RNase A (100 μg/ml in 2×SSC) for one hour at 37°C. Chromosomes were denatured for 90 seconds in 70% formamide in 2×SSC at 75°C. Labelled BACs were mixed with hybridization buffer and chicken genomic DNA or herring sperm DNA (Sigma-Aldrich Company Ltd., Dorset, UK) in a 1:2:1 ratio, applied to slides and sealed under cover slips. Hybridization was carried out in a humidified chamber at 37°C (overnight for same-species hybridizations and for three days for cross-species hybridizations (see below)). Post-hybridization washes for same-species hybridizations consisted of 50% formamide in 2 × SSC for 2 × 10 minutes at 37°C; one minute in 2 × SSC with 0.1% Igepal at RT; 15 minutes in 4 × SSC with 0.05% Igepal at RT; 25 minutes in 4 × SSC with 0.05% Igepal and 2% BSA at RT. For cross-species hybridizations, the first washing step was modified (10% formamide in 2 × SSC for 2 × 10 minutes at 30°C). Probes were detected with 1:200 streptavidin-Cy3 (Amersham, Little Chalfont, UK), in 4 × SSC, 0.05% Igepal, 1.25% BSA, plus 1:200 FITC-anti-digoxigenin (Amersham) for dual-color experiments, for 35 minutes at 37°C. Excess detection mix was removed by washing the slides in 4 × SSC, 0.05% Igepal for 3 × 3 minutes. Slides were counterstained using Vectashield with DAPI (Vector Labs, Burlingame, CA, USA). Slides were viewed using an Olympus BX-61 epifluorescence microscope equipped with a cooled CCD camera and appropriate filters. Images were captured using SmartCapture 3 (Digital Scientific, Cambridge, UK).

We also performed cross-species FISH experiments to investigate whether MHC-containing chromosomes in the zebra finch correspond to chicken chromosome 16 (Additional file 7). These experiments involved co-hybridization of zebra finch BACs *TGAC-102M22 *or *TGAC-86I22 *in combination with chicken BAC *WAG65G9 *(containing genetic markers *LEI0258 *and *MCW0371*) to chicken and zebra finch chromosomes. Unfortunately, none of these experiments gave unequivocal evidence for colocalisation of chicken and zebra finch MHC BACs and therefore the data are not shown.

### Gene and polymorphism survey via RFLP/Southern Blot

Restriction Fragment Length Polymorphisms (RFLP) were used to approximate the number of alleles for MHC Class I and Class IIB genes. We used the restriction enzyme Pvu II and digested seven micrograms of genomic DNA from ten captive zebra finches. These samples were run in two identical parallel agarose gels that were transferred to nylon membranes and then hybridized with radioactively labeled zebra finch class I and II probes, respectively (for details on southern blot see Westerdahl et al. [44]. The probes were prepared as follows; An MHC class I/IIB PCR product was cloned into a bacterial vector (TOPO-TA cloning kit, Invitrogen, Carlsbad, CA, USA inserts from five positive colonies were amplified and sequenced on a capillary sequencer according to manufacturer's protocol (Big Dye Terminator mix V3.1, Applied Biosystems, USA) and finally one MHC class I and one IIB insert, respectively, was amplified, cleanedand used as probes. The class I probe is a 271 bp exon 3 zebra finch DNA fragment (including primers), from a single colony, and it was amplified using the passerine Class I primers *PcaH1grw *(*5' -TCC CCA CAG GTC TCC ACA CMA T - 3'*) and *A23H3 *(*5' -TTG CGC TCY AGC TCY YTC YGC C - 3'*) using standard PCR conditions. The zebra finch class IIB probe covers 207 bps in exon 2 and it was amplified, from a single colony, using the primers *2zffw1 *(*5' - TGT CAC TTC AYK AAC GGC ACG GAG - 3'*) and *2zfrv1*(*5' - GTA GTT GTG CCG GCA GTA CGT GTC 3'*). The probes were labelled with (a-32P)dCTP (PerkinElmer Boston, MA, USA) using the nick-translation technique (GE-healthcare, Little Chalfont, UK)

### Comparative analysis of MHC genes

We estimated and compared gene density, across three avian lineages (chicken, quail and zebra finch) and the human *MHC*. To describe the human MHC, gene coordinates for protein coding genes were extracted from Ensembl [70] using the extended version of the human MHC map [79] as a template. To make an appropriate comparison among species, gene sets from human MHC were defined based on the flanking genes *SCGN *and *SYNGAP1*, but excluding pseudogenes, histones, tRNAs, vomeronasal and olfactory receptors. These genes were excluded because of their absence in the chicken *MHC *[20] and/or their lack of synteny with other MHC gene clusters in the zebra finch. For the chicken MHC gene set was based on the chicken extended *MHC *haplotype [20] and include the *MHC-Y *region. Gene coordinates were extracted using the latest annotation and assembly published in NCBI. For the quail *MHC*, extended regions have not been characterised and could not be included [35]. We calculated gene density by dividing the total number of genes by the total extent of the MHC region as defined above. Because zebra finch genes were often unmapped, and because of numerous pseudogenes, we approximated gene density for zebra finch using the two Class I BACs which appear to be a classical *MHC *region. Inclusion of all seven of the BACs also does also not alter the conclusions of this analysis. Although the zebra finch *MHC *assembly remains fragmented, the patterns revealed by this analysis highlight marked differences between zebra finch and chicken.

### Phylogenetic analysis and tests for selection

*Class I *(exon 3) and *Class IIB *(exons 2 and 3) sequences were downloaded from GenBank (Accession #s given in Figure 1). Nucleotide sequences were aligned using MUSCLE [80] and then imported into Se-Al [81] for manual verification. Sequences were translated into amino acids and then adjusted by eye. All phylogenetic analyses were done using MrBayes v 3.1.2 [82]. For *Class IIB *we analyzed the two exons separately (not shown) and in a combined analysis where the data were partitioned by exon, and models were fitted to each codon position independently. To determine an appropriate root for passerine *MHC *sequences we first conducted analyses across all birds (including raptors, galliforms, and shorebirds, not shown here). For use in tests of selection, we conducted further analyses using only passerine sequences. MrBayes was run for 2.4 million generations, with 400,000 generations discarded as burn-in. One thousand sampled trees were then used to generate consensus trees and posterior probabilities. Trees from MrBayes and sequence alignments were analyzed in PAML [49,50] to test for evidence of selection acting on sites in the alignments. We used CODEML and tested two pairs of models using likelihood ratio tests. We tested the M1 model of nearly neutral evolution versus the M2 model of positive selection. We also tested the M7 model with the M8 model in which ω (d_N_/d_S_) can be greater than one. Both of these tests are routinely used to test for the influence of positive selection. Bayes Empirical Bayes analyses was used to identify specific sites with ω > 1 [83]. We also constructed phylogenies using only chicken and zebra finch sequences, but including putative zebra pseudogenes that spanned the exons of interest.

## Abbreviations

BAC: bacterial artificial chromosome; Cds: coding sequence; ERV: endogenous retrovirus; EST: expressed sequence TAG; FISH: fluorescence in situ hybridization; LTR: long terminal repeat; RFLP: restriction fragment length polymorphism; UTR: untranslated region.

## Authors' contributions

CNB and SVE designed the study. CNB screened the BAC library, annotated BAC clones and conducted phylogenetic analyses. RE and RG assessed the genome assembly. HK, TG and WW sequenced and assembled the BAC clones. RE and RG assisted with the annotation of BAC clones. HW conducted the RFLP analysis. MV and DG conducted the FISH mapping. DB sequenced plasmids used in the BAC assemblies. All authors contributed to the writing and/or the editing of the manuscript.

## Supplementary Material

Additional file 1 **Overgo probes used for BAC library screening**. Overgo probes targeting five genes of the MHC. Two pairs of probes were designed for each gene using sequences from the zebra genome trace archive. Click here for file

Additional file 2 **Self-self BLAST analysis of six BAC assemblies (Class II: A to D, Class I: E to F)**. Theses results highlight the repetitive nature of these genomic regions, and the challenges faced in assembly. Click here for file

Additional file 3 **Genes found by BAC sequencing**. Genes found by BAC sequencing and manual and automated gene prediction. Click here for file

Additional file 4 **Two-color FISH mapping of TAP2 and MHC Class I BACs**. Depicted is the only case in which BACs putatively containing TAP2 and Class I colocalised. Colocalisation was on the W chromosome. Click here for file

Additional file 5 **BACs used in two color FISH mapping**. BACs used in dual-color FISH experiments with zebra finch MHC. These BACs are specific for zebra finch microchromosomes 9-15 and 17-28. None of these BACs cohybridized with MHC BACs. Because the whole genome assembly places some MHC genes on chromosome 22, we tested two chromosome 22 BACs. Both of these cohybridize with each other, and neither cohybridized with MHC BACs. Click here for file

Additional file 6 **Phylogenetic analysis and selection on *MHC Class I *sequences**. **A) **Phylogenetic relationships among passerine MHC Class I, exon 3 sequences. Only one sequence with open reading frames were found in the zebra finch genome. The remaining sequences are from GenBank. **B) **Predicted amino acid sequences of the genomic sequence and one EST for MHC Class I. Stars represent sites showing evidence of selection in passerine birds. Note the similarity in the selected sites between raptors and passerines, both of which correspond well with the human PBR. Click here for file

Additional file 7 **Preparation of chicken chromosomes**. The method for the preparation of chicken chromosome spreads is described. Click here for file
