# Supplementary material for: Gene duplication and fragmentation in the zebra finch major histocompatibility complex
Source: BMC Biol. 2010 Apr 1;8:29. doi: 10.1186/1741-7007-8-29 (PMC2907588; doi:10.1186/1741-7007-8-29)
Supplement: Additional file 1 — Overgo probes used for BAC library screening. Overgo probes targeting five genes of the MHC. Two pairs of probes were designed for each gene using sequences from the zebra genome trace archive. [file 1741-7007-8-29-S1.PDF]

Additional File 1. Overgo probes designed to target five genes of the MHC. Two pairs of probes were designed for each gene using sequences from the zebra genome trace archive.

Gene	Overgo A	Overgo B
KIFC1	GCTGTTCCTGTATGGAATGTGTGG	CCTCTGCAATAAGGAGCCACACAT
	CTCGTAGTAGATCTCCAGGAAGCT	TACGGCTTATCTGCCAGCTTCCT
Class I	CTTGGGAACACGGTAATTCCTGTG	CTACCCTTGGAATTCCCACAGGAA
	ATTTCACTCTCATCCCATCTCAGT	ACCATGGAATTCCCACTGAGAT
TNXB	CTCCTTTGACTCCTTCTTGCTGCA	ATGGACATCCCCGTACTGCAGCAA
	TTGACTCCTTCTTGCTGCAGTAC	CCATGGACATCCCCGTACTGCA
TAP2	TTGCGGTGCAGGTACTGGTGTTGG	TCCCGCTACCAGAATACCAACACC
	AGGTACTGGTGTTGGTATTCTGGT	GATGGGATCCCGCTACCAGAAT
CENPA	AGCGAGCAGAGGTACGCGTCTTCC	TCATCGTGCGGATGCTGGAAGACG
	AGAGGTACGCGTCTTCCAGCAT	CCTTCATCGTGCGGATGCTGGA
