# Supplementary figures and images for: Gene duplication and fragmentation in the zebra finch major histocompatibility complex
Source: BMC Biol. 2010 Apr 1;8:29. doi: 10.1186/1741-7007-8-29 (PMC2907588; doi:10.1186/1741-7007-8-29)

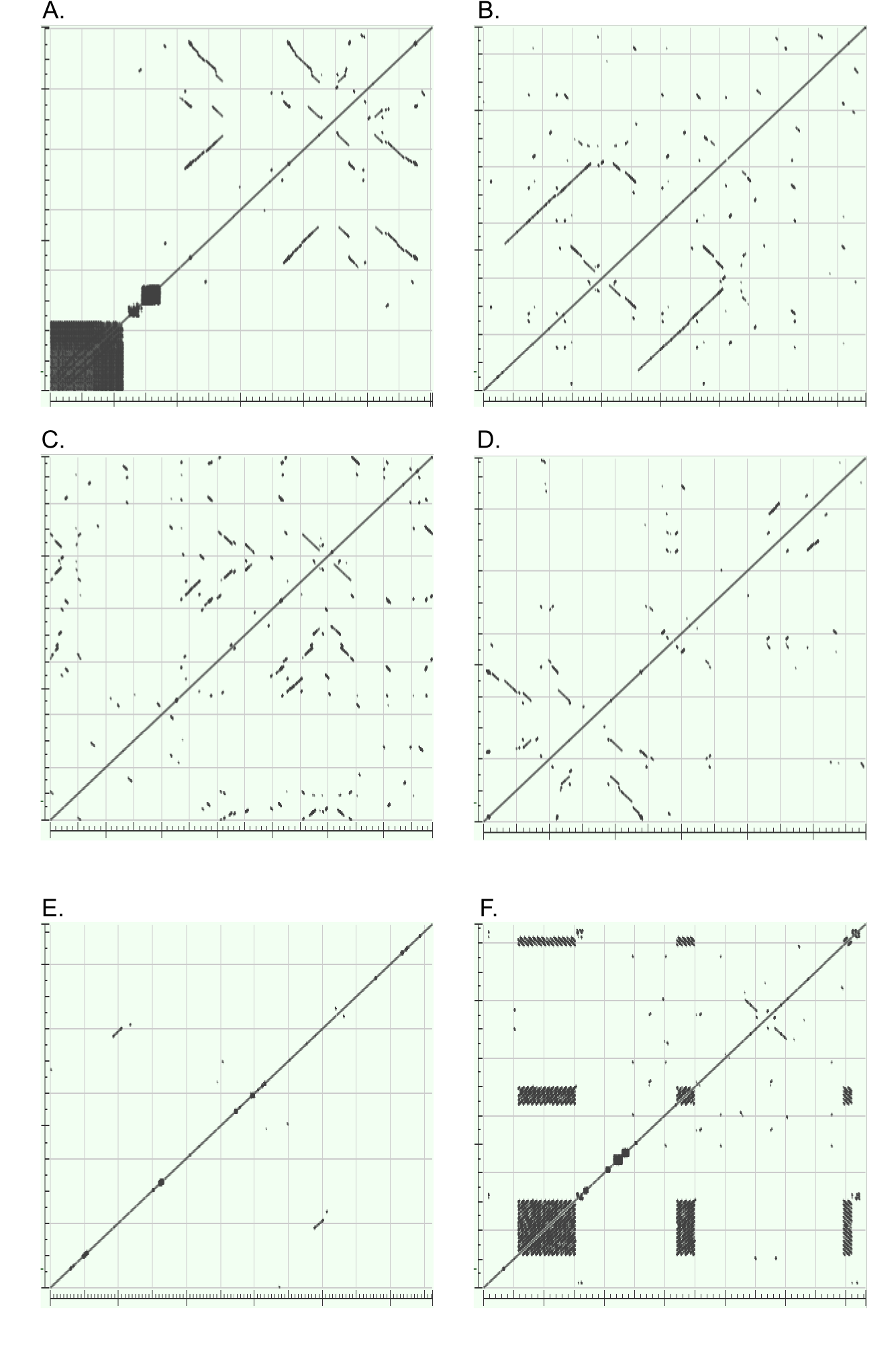

Supplement: Additional file 2 — Self-self BLAST analysis of six BAC assemblies (Class II: A to D, Class I: E to F). Theses results highlight the repetitive nature of these genomic regions, and the challenges faced in assembly. [file 1741-7007-8-29-S2.PNG]

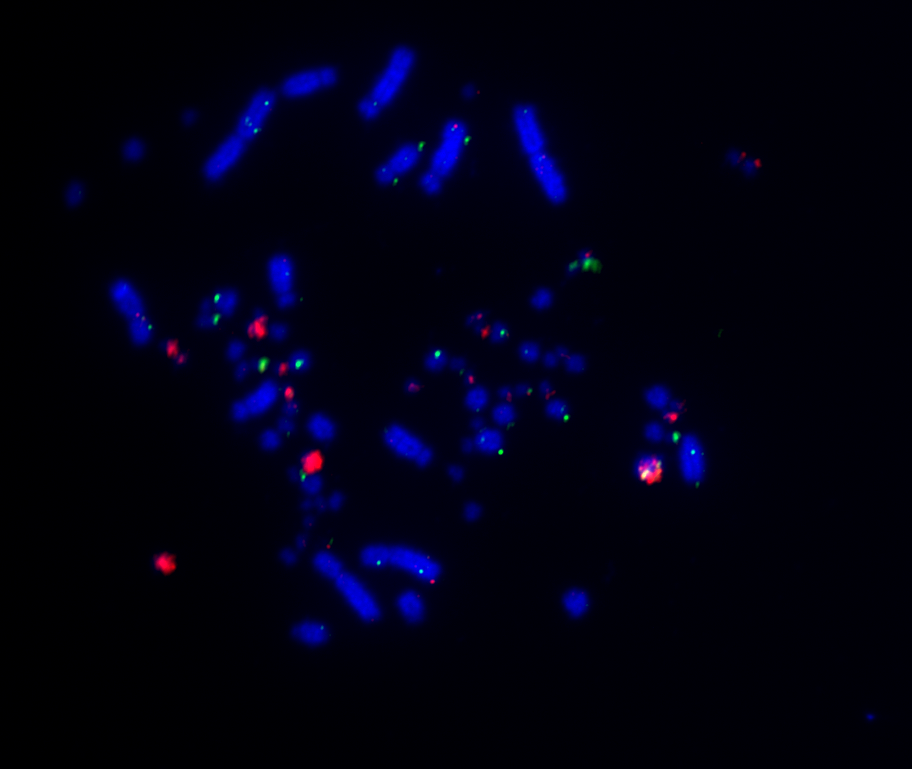

Supplement: Additional file 4 — Two-color FISH mapping of TAP2 and MHC Class I BACs. Depicted is the only case in which BACs putatively containing TAP2 and Class I colocalised. Colocalisation was on the W chromosome. [file 1741-7007-8-29-S4.TIFF]

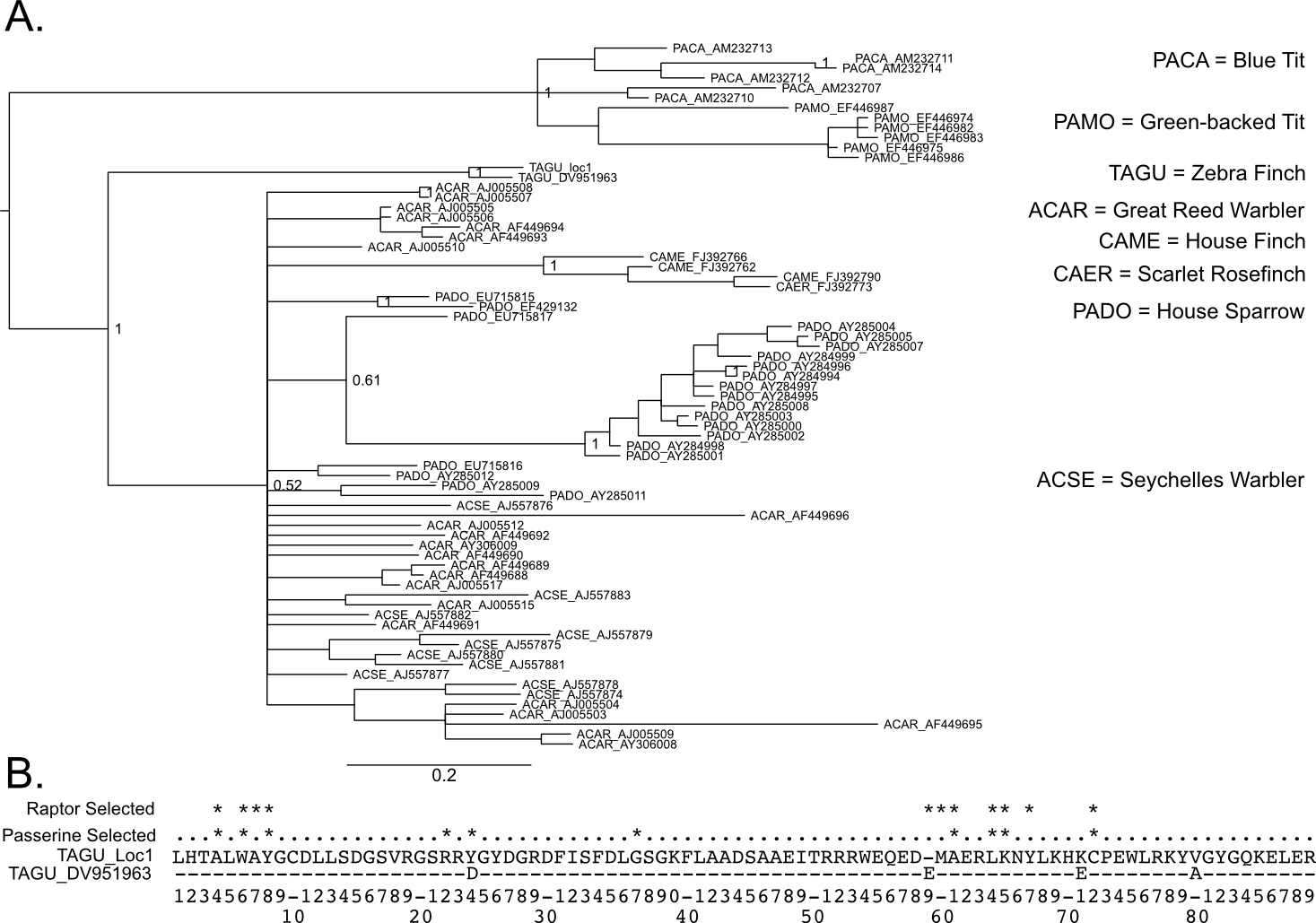

Supplement: Additional file 6 — Phylogenetic analysis and selection on MHC Class I sequences. A) Phylogenetic relationships among passerine MHC Class I, exon 3 sequences. Only one sequence with open reading frames were found in the zebra finch genome. The remaining sequences are from GenBank. B) Predicted amino acid sequences of the genomic sequence and one EST for MHC Class I. Stars represent sites showing evidence of selection in passerine birds. Note the similarity in the selected sites between raptors and passerines, both of which correspond well with the human PBR. [file 1741-7007-8-29-S6.PNG]
