# Supplementary material for: Gene duplication and fragmentation in the zebra finch major histocompatibility complex
Source: BMC Biol. 2010 Apr 1;8:29. doi: 10.1186/1741-7007-8-29 (PMC2907588; doi:10.1186/1741-7007-8-29)
Supplement: Additional file 3 — Genes found by BAC sequencing. Genes found by BAC sequencing and manual and automated gene prediction. [file 1741-7007-8-29-S3.PDF]

Additional File 3. Genes found in BACs by automated and manual gene predictions.

Gene	BAC Clone	Notes and relationship w/ genome assembly
MHC Class IIB	TGAA-157B03	Portion of exon 3, identical to ΨM
	TGAA-157B03	Exons 1-3, identical to ΨT
	TGAA-323J16	Exon 1 (and possibly 4), similar to Loc2
	TGAA-323J16	Exon 4. Not similar to others in genome
	TGAA-323J16	Exons 3-4. Similar to ΨQ from the genome
	TGAA-323J16	Exons 3-4. Identical to above.
	TGAA-323J16	Full gene (6 exons). Identical to Locus 2
	TGAA-351E14	Full gene (6 exons). Identical to Locus 2
	TGAA-351E14	Exon 4. Not similar to others in genome
	TGAA-351E14	Exons 3-6. Likely pseudogene. unique.
	TGAA-47O03	Exons 1-3. Identical to ΨT
	TGAA-47O03	Exons 1 and 3. Identical to ΨM
	TGAA-47O03	Exons 1 and 4. Similar to ΨM
TAPBP	TGAA-157B03	Exons 1-4
TAP1	TGAC-167E04	Full gene (8 exons).
TAP2	TGAC-167E04	Exons 1 and 6.
TNXB	TGAC-167E04	3 exons (matching parts of exons 8-11 in chicken)
MHC Class I	TGAC-102M22	Full gene (8 exons). Similar to Locus 1
DAXX	TGAC-102M22	3 exons (matching parts of exons 2 and 3 in human)
FLOT1	TGAC-102M22	Full gene (12 exons)
KIFC	TGAC-102M22	Exons 3-10
TUBB	TGAC-102M22	Exons 3-4
