# Supplementary material for: Gene duplication and fragmentation in the zebra finch major histocompatibility complex
Source: BMC Biol. 2010 Apr 1;8:29. doi: 10.1186/1741-7007-8-29 (PMC2907588; doi:10.1186/1741-7007-8-29)
Supplement: Additional file 5 — BACs used in two color FISH mapping. BACs used in dual-color FISH experiments with zebra finch MHC. These BACs are specific for zebra finch microchromosomes 9-15 and 17-28. None of these BACs cohybridized with MHC BACs. Because the whole genome assembly places some MHC genes on chromosome 22, we tested two chromosome 22 BACs. Both of these cohybridize with each other, and neither cohybridized with MHC BACs. [file 1741-7007-8-29-S5.PDF]

Addtional File 4. BACs used in dual-color FISH experiments with zebra finch MHC BACs. These BACs are specific for zebra finch microchromosomes 9-15 and 17-28. None of these BACs cohybridized with MHC BACs. Because the assembly places some MHC genes on chromosome 22, we tested two chromsome 22 BACs. Both of these cohybridize with each other, and neither cohybridized with MHC BACs.

BAC code	Chromosome
TGAC-217A03	9
TGAC-310P11	10
TGAC-105N23	11
TGAC-342P15	12
TGAC-178L17	13
TGAC-45H05	14
TGAC-135G20	15
TGAC-197G19	17
TGAC-263I20	18
TGAC-84A03	19
TGAC-225I12	20
TGAC-119I06	21
TGAC-113N13	22
TGAC-151I22	22
TGAC-272G09	23
TGAC-321M03	24
TGAC-65M01	25
TGAC-232I02	26
TGAC-23C05	27
TGAC-37M13	28
