# Supplementary material for: Gene duplication and fragmentation in the zebra finch major histocompatibility complex
Source: BMC Biol. 2010 Apr 1;8:29. doi: 10.1186/1741-7007-8-29 (PMC2907588; doi:10.1186/1741-7007-8-29)
Supplement: Additional file 7 — Preparation of chicken chromosomes. The method for the preparation of chicken chromosome spreads is described. [file 1741-7007-8-29-S7.PDF]

Methods

Chicken chromosomes were prepared from lymphoblast cultures. Lymphocytes were isolated from whole blood using Histopaque (Sigma) according to the manufacturer's instructions and cultured at 37ºC, 5% CO_2 in RPMI1640 medium with a final concentration of 20mM L-glutamine, 0.1mg/ml concanavalin A type IV (Sigma), 10% chicken serum (Invitrogen) and standard antibiotics (penicillin/streptomycin, Invitrogen). After 72 hours, cultures were treated with colcemid at a final concentration of 0.05µg/ml for 45 minutes at 37ºC, followed by hypotonic treatment with 75mM KCl for 15 minutes at 37ºC and fixation in 3:1 methanol:acetic acid.(mapping was done as in main text)
